# Downregulation of lncRNA MEG3 and miR-770-5p inhibit cell migration and proliferation in Hirschsprung’s disease

**DOI:** 10.18632/oncotarget.19207

**Published:** 2017-07-12

**Authors:** Hongxing Li, Bo Li, Dongmei Zhu, Hua Xie, Chunxia Du, Yankai Xia, Weibing Tang

**Affiliations:** ^1^ Department of Pediatric Surgery, Children’s Hospital of Nanjing Medical University, Nanjing, China; ^2^ State Key Laboratory of Reproductive Medicine, Institute of Toxicology, School of Public Health, Nanjing Medical University, Nanjing, China; ^3^ Key Laboratory of Modern Toxicology, Nanjing Medical University, Ministry of Education, Nanjing, China; ^4^ Department of Hepatobiliary Surgery, The Second Affiliated Hospital of Nantong University, Nantong, China

**Keywords:** lncRNA, miRNA, gene regulation, Hirschsprung’s disease

## Abstract

The long noncoding RNA (lncRNA) MEG3 is involved in various biological processes including cell migration and cell proliferation. In present study, it was found that MEG3 and the intronic miR-770-5p were decreased in samples from HSCR patients. Besides, knockdown of MEG3 and miR-770-5p suppressed cell migration and proliferation, while cell cycle and apoptosis were not affected in human 293T and SH-SY5Y cells. SRGAP1 mRNA and protein upregulation was inversely correlated with miR-770-5p expression in tissue samples and cell lines, which was confirmed to be a target gene of miR-770-5p by dual-luciferase reporter assay. Moreover, silencing of SRGAP1 rescued the inhibition of cell migration and proliferation induced by MEG3 siRNA and miR-770-5p inhibition. The present study elucidates a novel mechanism of the development of HSCR and shows that the MEG3/miR-770-5p/SRGAP1 pathway plays a vital role in the pathogenesis of HSCR.

## INTRODUCTION

Hirschsprung‘s disease (HSCR), a congenital enteric disease, is characterized by the absence of ganglion cells from various regions of the distal bowel caused by defects in the migration of neural crest-derived progenitor cells during embryogenesis [1, 2]. The deficiency of enteric neurons leads to abnormal gut motility, which is manifested by a delay in meconium discharge, abdominal distension, and intestinal obstruction. The incidence of HSCR is approximately 1:5000 live births with a 4:1 male:female gender bias [3]. Approximately 18% of HSCR patients show a syndrome in which HSCR is only a constituent part [4]. To date, many genes have been associated with the pathogenesis of HSCR, including RET, GDNF, NRG1, EDNRB, SOX10 and so on [5–7]. Furthermore, the role of non-coding RNAs including long non-coding RNAs (lncRNA) and short non-coding RNAs (microRNA) have been expound to be involved in the appearance of HSCR [8, 9].

lncRNAs, which are more than 200 nucleotides in length and do not encode proteins, also play an important role in physiological development, especially in the cancer disease [10]. What’s more, study has indicated that loss of lncRNAs function leads to neural disease [11]. However, miRNAs, which are approximately 20–24 nucleotides in length, regulate gene expression post-transcriptionally by interacting with the 3′ untranslated region (3′-UTR) of target genes [12]. miRNAs play roles in energy homeostasis, especially the regulation of intestinal homeostasis, cell migration, cell cycle, cell apoptosis, cell proliferation, and neuronal function [13–15]. Previous studies from our group have been focusing on the effect of miRNAs on the development of HSCR [16, 17].

The lncRNA MEG3, which acts as tumor suppressor, is a precursor of miR-770-5p and plays critical roles in many diseases [18, 19]. Recent date indicated that lncRNA H19 was abnormally increased and contributed to the cell proliferation via miR-675 in gastric cancer [20]. Nevertheless, the relationship between MEG3, miR-770-5p and the role of MEG3 in HSCR have not been reported. Recently, the study of interaction between lncRNA and miRNA has become a hot topic. There are several main pathways mediating the regulation of miRNAs by lncRNAs. Firstly, lncRNAs indirectly inhibit miRNA negative gene regulation by competing for binding to the 3′-UTR of target genes [21]. Secondly, lncRNAs serve as competitive endogenous gene regulating miRNAs and further impacting cell cytobiology [22, 23]. Thirdly, some lncRNAs are similar to protein-coding genes that act as host genes and can be processed to miRNAs, which then function to modulate the target gene [24]. The last intron of MEG3 encodes miR-770-5p, which is expressed in senior mammals such as humans, horses, Rhesus monkeys, chimpanzees, the house mouse, and Norway rat. Bioinformatics analyses indicate that it may have important biological effects. Given the role of lncRNAs in neural diseases, the importance of the relation between lncRNAs and miRNAs in the pathophysiologic mechanism, and the MEG3 and miR-770-5p were abnormally decreased in HSCR, we speculated that MEG3, miR-770-5p may be involved in the pathogenesis of HSCR.

Thus, we investigated the functional involvement of MEG3 and its intron miR-770-5p in HSCR progression and also identified the target gene of the miR-770-5p, SRGAP1, which plays important roles in the development of the nervous system. In this study, we explored the mechanism of pathophysiological roles and the potential relationship between lncRNA MEG3 and miR-770-5p, indichating that the lncRNA MEG3/miR-770-5p/SRGAP1 pathway may play important roles in the pathogenesis of HSCR.

## RESULTS

### Clinical information analysis

The present study analyzed 192 human colon tissue specimens obtained from the Department of Pediatric Surgery, Children’s Hospital of Nanjing Medical University, including 96 HSCR-confirmed cases and 96 matched controls. Clinical information including age, gender (male/female), and body weight were obtained from participants. There were no statistically significant differences in age (3.60± 0.22 and 3.33 ± 0.25 months old) and body weight (5.2 ± 0.32 and 4.8 ± 0.26 kg) between HSCR and controls. The gender rate (Male/Female) of HSCR and controls was 78/18 and 71/25, respectively, which matched the gender rate of this disease.

### MEG3, the host gene of miR-770-5p, positively correlates with miR-770-5p expression in HSCR patients

**Figure 1:**
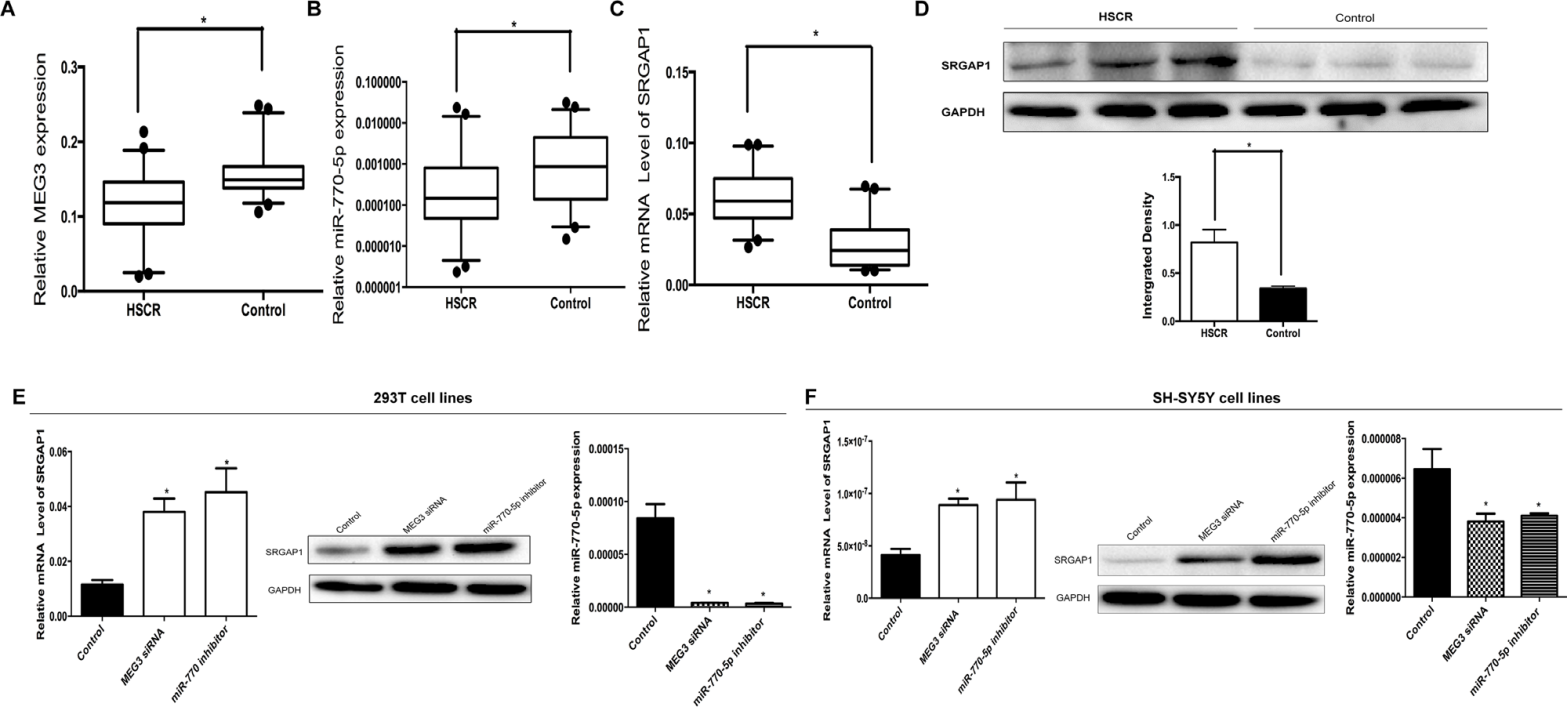
The lncRNA MEG3 positively correlates with miR-770-5p expression in HSCR patients **(A, B, C)** The lncRNA MEG3, miR-770-5p and SRGAP1 expression were detected in HSCR patient colon tissues (n=96) and controls (n=96). Data are presented as a box plot of the median and range of log-transformed relative expression level. **(D)** The protein level of SRGAP1 in HSCR tissues. **(E, F)** The 293T and SH-SY5Y cells were transfected with MEG siRNA and miR-770-5p inhibitor. The mRNA and protein expression of SRGAP1 and miR-770-5p were determined by qRT-PCR and Western blot.

miR-770-5p is located in the intron of the lncRNA MEG3. To determine the role of MEG3 and miR-770-5p in the occurrence of HSCR, we assessed the expression level of MEG3 and miR-770-5p. The results showed that MEG3 was downregulated in HSCR (Figure 1A) and consistent with the expression levels of miR-770-5p (Figure 1B), indicating that low-expression level of miR-770 may have an effect on the development of HSCR.

To further elucidate the relationship between MEG3 and miR-770-5p, we transfected the 293T and SH-SY5Y cells with MEG3 siRNA. It was found that deprivation of MEG3 expression remarkably reduced miR-770-5p expression (Figure 1E and 1F) in both cells, which indicating that miR-770-5p was regulated by MEG3.

### MEG siRNA reduced cell migration and proliferation, whereas overexpression of MEG3 promoted cell migration and proliferation

**Figure 2:**
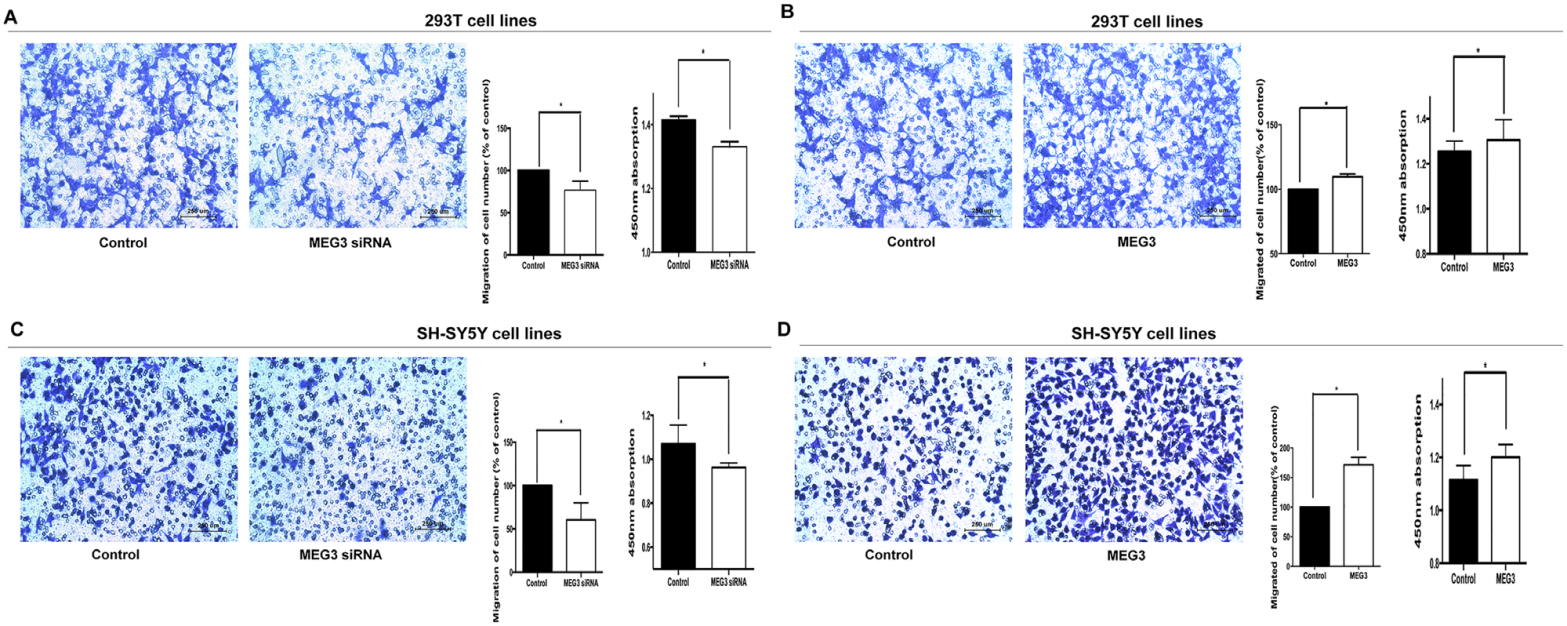
The cellular function of MEG3 in 293T and SH-SY5Y cells **(A, B)** MEG3 siRNA and overexpression lentivirus were transfected into 293T cells and cultured in transwell, Migrated cells were then colored with violet crystal and counted. The relative quantity of migrated cells is presented as a percentage of total cells (right). Absorbance at 450 nm is presented as mean ± SE. **(C, D)** The Cytobiology change after treating SH-SY5Y cellsw ith MEG3 siRNA and overexpression lentivirus.* means significant difference (P < 0.05).

Cell migration and proliferation assays were performed to verify the function of MEG3. MEG3 siRNA was transfected into 293T and SH-SY5Y cells. Results showed that knockdown of MEG3 inhibited cell migration and proliferation (Figure 2A and 2C). Meanwhile, cell cycle progression and apoptosis were not affected by MEG3 siRNA transfection (Supplementary Figure 1).

MEG3 was overexpressed by infecting 293T and SH-SY5Y cells with a MEG3-overexpression lentivirus, and cell migration and proliferation were examined after 72 hours. As shown in (Figure 2B and 2D), overexpression of MEG3 increased cell migration and proliferation. However, MEG3 overexpression had no effect on cell cycle progression and cell apoptosis (Supplementary Figure 1).

### miR-770-5P inhibitor suppressed cell migration and proliferation

**Figure 3:**
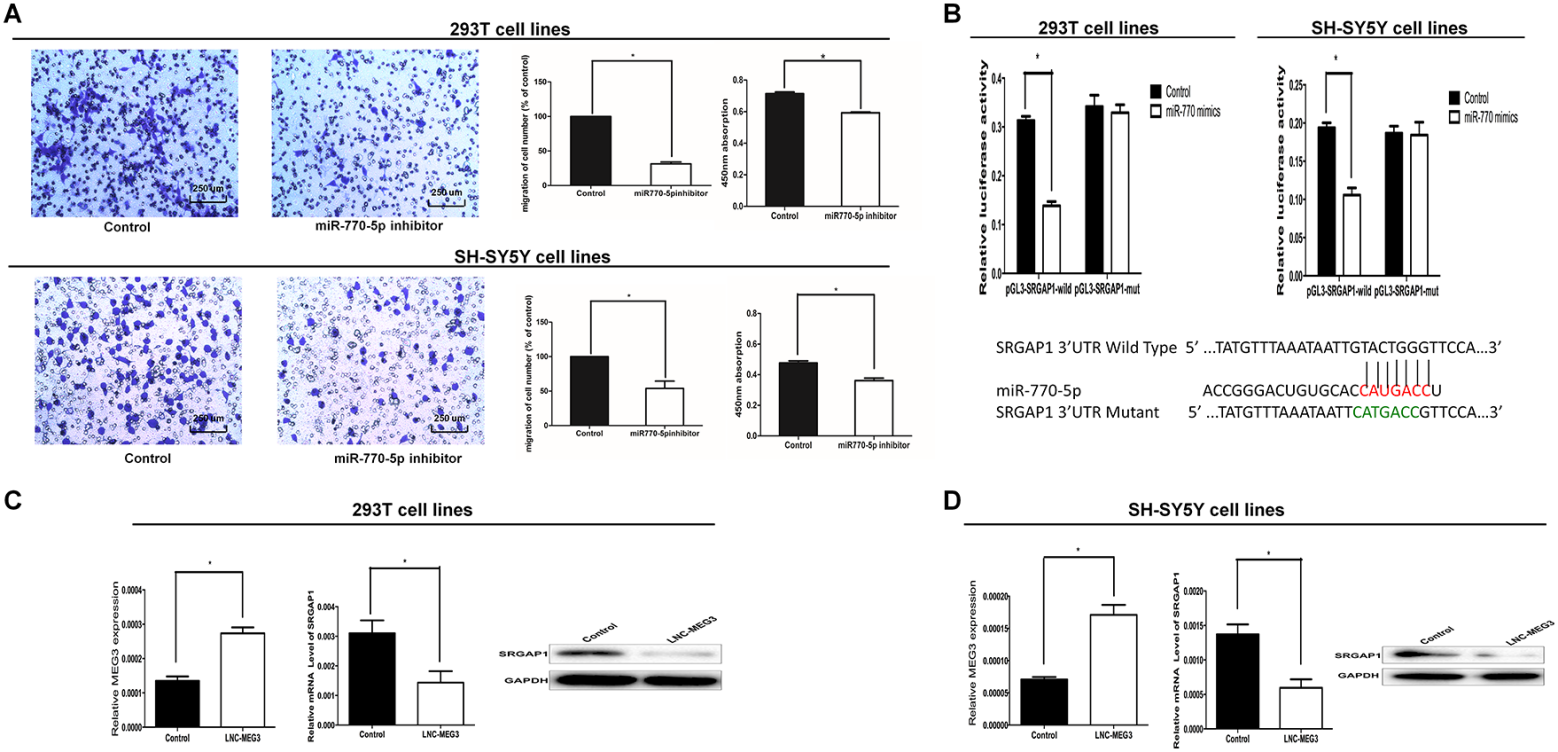
SRGAP1 is a downstream target of MEG3/miR-770-5p **(A)** Cell migration and proliferation were significantly decreased in 293T and SH-SY5Y cells transfected with the miR-770-5p inhibitor. **(B)** Bottom: Sequence alignment of human miR-770-5p with 3’-UTR of SRGAP1. Mutations in the 3’-UTR of SRGAP1. TOP: The firefly luciferase activity in 293T and SH-SY5Y cells after cotransfection with reporter construct and miR-770-5p mimics. **(C, D)** The mRNA and protein expression of SRGAP1 was determined by qRT-PCR and Western blot after transfected with MEG3-overexpression lentivirus in 293T and SH-SY5Y cells. All results were presented as mean ± SE.* means significant difference (P < 0.05).

To confirm the cellular function of miR-770-5P, we examined the effect of miR-770-5p on cell migration, cell proliferation, cell cycle procession, and apoptosis. The Transwell assay and CCK8 assay were used to test cell migration and cell proliferation in 293T and SY5Y cells. Results showed that cell migration and proliferation were significantly decreased in cells transfected with the miR-770-5p inhibitor, suggesting that inhibition of miR-770-5p suppresses cell migration and proliferation (Figure 3A). Flow cytometric analysis was performed to test the role of miR-770-5p in cell cycle progression and apoptosis. However, there were no differences in cell cycle progression or the percentage of apoptotic cells between miR-770-5p inhibitor transfected and control cells (Supplementary Figure 1).

### SRGAP1 was the target gene for miR-770-5P

Three databases (MICRORNA.ORG, TRAGETSCAN and MIRDB) were used to predict target genes of miR-770-5p. Three candidate target genes, CCND2 (cyclin D2), STK38L (serine/threonine kinase 38 like), and SRGAP1 (SLIT-ROBO Rho GTPase activating protein 1), were selected after the prediction and functional analysis. To determine whether the three predicted target genes have connections with HSCR, qRT-PCR was used to measure the mRNA levels in 96 HSCR colon tissues and 96 matched controls. SRGAP1 expression was upregulated in cases compared with controls (Figure 1C). However, no differences of CCND2 and STK38L expression levels were detected between cases and controls. The protein levels of SRGAP1 were consistent with the mRNA levels (Figure 1D).

To confirm that SRGAP1 is a target gene for miR-770-5p, two independent methods were used. Firstly, the wild-type or a mutant seed sequence of SRGAP1 that was shared by miR-770-5p was cloned into the pLG3 vector to yield pLG3-SRGAP1 or pLG3-SRGAP1-mut. The relative luciferase activity of the group co-transfected with PLG3-SRGAP1 and miR-770-5p mimics was significantly decreased (Figure 3B). In contrast, no difference between the luciferase activity of PLG3-SRGAP1-mut and the negative control was observed. These results indicated that miR-770-5p decreased SRGAP1 expression by binding to the 3′-UTR of SRGAP1. Secondly, the SRGAP1 mRNA and protein levels were evaluated by qRT-PCR and western blotting separately after transfected with miR-770-5p inhibitor and control in 293T and SH-SY5Y cells for 48 h. SRGAP1 expression was significantly increased at both mRNA and protein levels in 293T and SH-SY5Y cells (Figure 1E and 1F).

### MEG3 effect on cell migration and proliferation was mediated by the miR-770-5p/SRGAP1 pathway in HSCR

**Figure 4:**
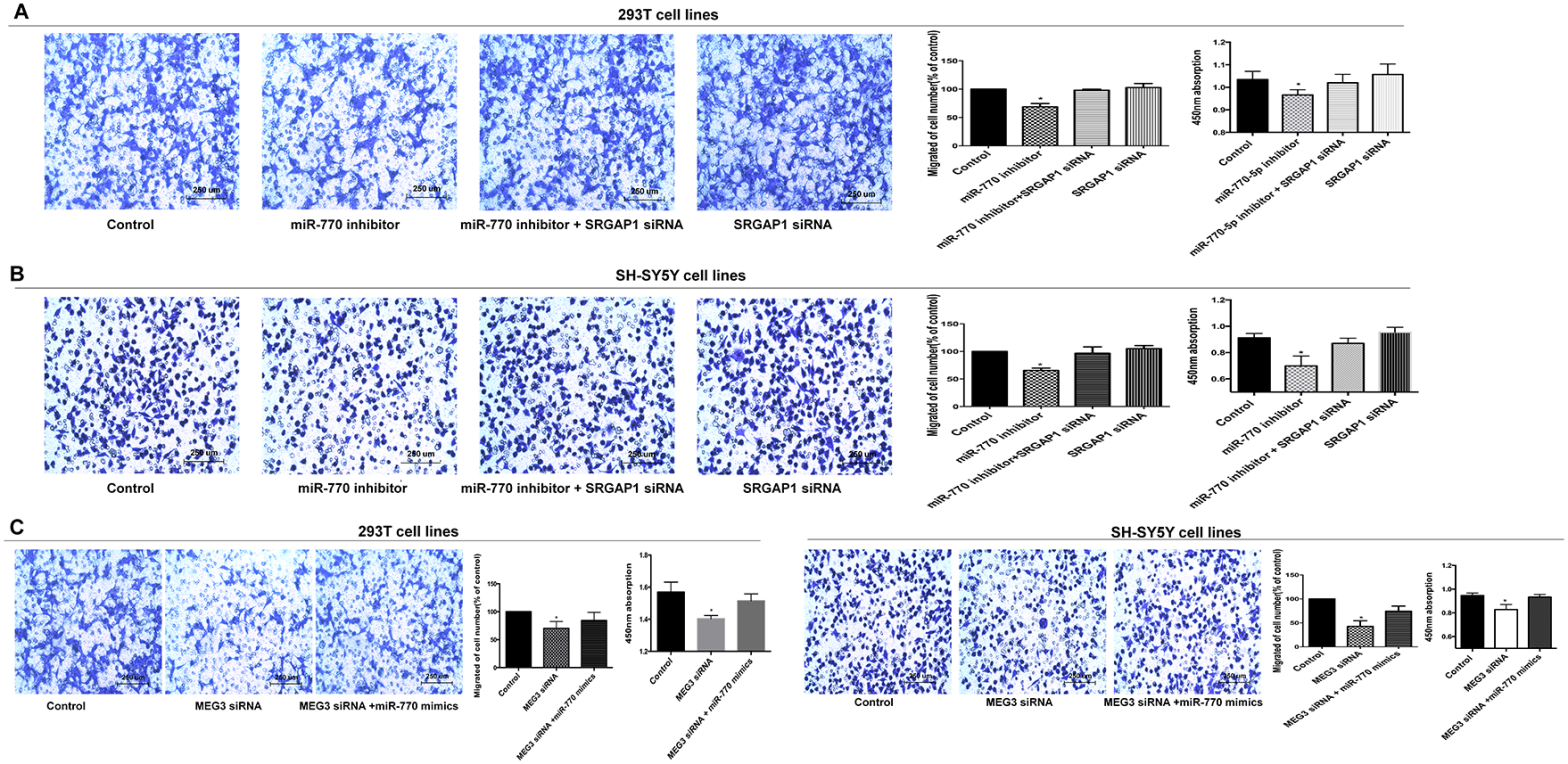
MEG3 effect on cell migration and proliferation was mediated by the miR-770-5p/SRGAP1 pathway in HSCR **(A, B)** SRGAP1 siRNA could partly reverse cell migration and proliferation in 293T and SH-SY5Y cell lines transfected with miR-770-5p inhibitor. **(C)** Co-transfection of miR-770-5p mimics partially rescued the MEG3 siRNA-mediated decrease in cell migration and proliferation. Absorbance at 450 nm was presented as mean ± SE. * means significant difference (P < 0.05).

To determine whether MEG3 functions via the miR-770-5p/SRGAP1 pathway, we firstly transfected 293T and SH-SY5Y cells with MEG3 siRNA. miR-770-5p expression was downregulated, whereas SRGAP1 was upregulated at the mRNA and protein levels (Figure 1E and 1F). Secondly, infection of the two cell lines with MEG3-overexpression lentivirus resulted in increased MEG3 expression levels, whereas SRGAP1 expression was downregulated at the mRNA and protein levels (Figure 3C and 3D). Thirdly, cell migration and proliferation assays were used to explore functional changes when the two cell lines were co-transfected with MEG3 siRNA and miR-770 mimics, MEG3-overexpression lentivirus, and miR-770 inhibitor singly. Co-transfection of miR-770-5p mimics partially rescued the MEG3 siRNA-mediated decreasing in cell migration and proliferation (Figure 4C). Taken together, these results suggested that miR-770-5p is a pivotal mediator in MEG3-induced HSCR progression.

**Figure 5:**
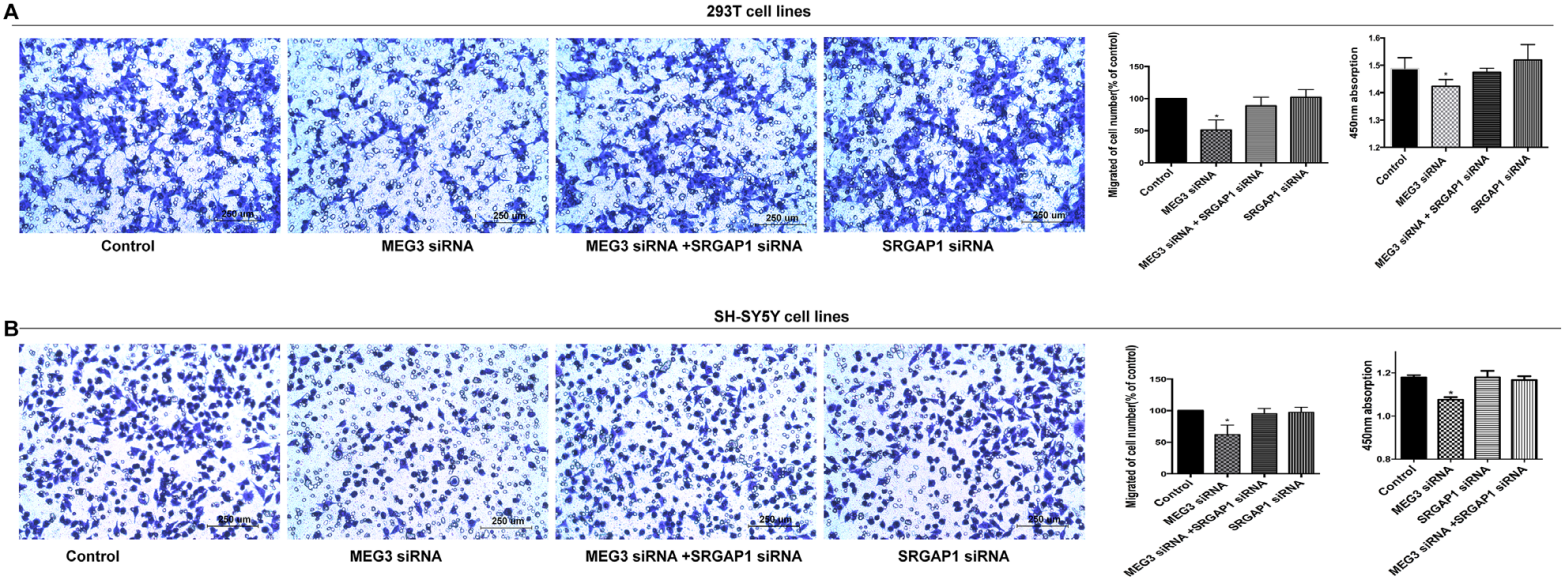
Migration and proliferation were reversed after co-transfection with MEG3 siRNA and SRGAP1 siRNA **(A, B)** SRGAP1 siRNA could partly reverse cell migration and proliferation in 293T and SH-SY5Y cell lines transfected with MEG3 siRNA. Absorbance at 450 nm was presented as mean ± SE. * means significant difference (P < 0.05).

To further examine whether SRGAP1 has an effect on cell migration and proliferation, we performed a series of rescue experiments in which 293T and SH-SY5Y cells were co-transfected with miR-770-5p inhibitor and SRGAP1 siRNA, and MEG3 siRNA and SRGAP1 siRNA separately. Cell migration was increased in response to SRGAP1 silencing compared with that in cells transfected with miR-770-5P inhibitor or MEG3 siRNA alone, although it was still lower than that of the control. Migration ability in cells transfected with SRGAP1 siRNA was similar to that of the control. Cell proliferation, as detected by the CCK8 assay, showed changes consistent with those observed in the cell migration assay (Figure 4A and 4B, Figure 5). These results clearly indicated that the effect of MEG3 on cell migration and proliferation was mediated by the miR-770-5p/SRGAP1 pathway in HSCR.

## DISCUSSION

HSCR, which is a congenital disease of the digestive tract in newborns, is characterized by the absence of ENS neurons originated from neural crest cells. ENS neurons fail to migrate and colonize the affected gut regions of variable lengths during embryogenesis from 5 to 12 weeks [25]. HSCR has multifactorial disorders and many genes, including noncoding RNAs, play roles in the pathogenesis of HSCR. HA117, as an ATRA-related lncRNA, was shown to be associated with the etiology of HSCR [9].

LncRNAs, which localize both to the nucleus and cytoplasm, play functional roles in the development and function of the nervous system [26]. In addition, lncRNAs are involved in various biological processes, including cell migration, cell proliferation, cell cycle progression, cell apoptosis, and cell differentiation. Recent studies show that lncRNAs and miRNAs that act as host genes have concordant expression, such as miR-31 and its host gene miR-31HG [27]. In the present study, MEG3 and miR-770-5p, which is located in its intronic region, were both downregulated in HSCR patients. We speculated that MEG3, miR-770-5p are involved in the pathogenesis of HSCR. however, the underlying mechanism of miR-770-5p and MEG3 is still unknown.

We firstly confirmed that MEG3 and miR-770-5p play a role in the etiology of HSCR. Moreover, assessment of RNA levels showed that the expression levels of miR-770-5p were consistent with MEG3 expression levels, indicating that MEG3 may function via miR-770-5p. In addtion, Cell migration and proliferation changes caused by MEG3 siRNA were partly rescued by miR-770-5p mimics, the same with the change in the overexpression of MEG3 as expected. Those results suggested that miR-770-5p is a pivotal mediator in MEG3-induced HSCR progression.

To validate SRGAP1 as the target gene of miR-770-5p, two cell lines were transfected with miR-770-5p inhibitor, and SRGAP1 mRNA and protein levels were found upregulated. In the following step, a dual-luciferase reporter assay was performed to confirm the relationship between miR-770-5p and SRGAP1. The results showed that SRGAP1 was the target gene of miR-770-5p.

SRGAP1 expressed in mammals, regulates cell migration by interacting with Robo1 and Rho GTPases, and plays important roles in the development of the nervous system [28]. SRGAP1 allows the concomitant activation of Rac1 and RhoA via its RacGAP activity to regulate cell migratory behavior. Continuous spatiotemporal spreading and a modal shift of intrinsic cell motility from random to directionally persistent result from depletion of SRGAP1, which overactivates Rac1 and inactivates RhoA [29]. In the present study, SRGAP1 was upregulated in HSCR patients. To examine the effect of SRGAP1 on cell migration and proliferation in HSCR, a series of rescue experiments were performed. As expected, SRGAP1 siRNA, when co-transfected with MEG3 siRNA or miR-770-5p inhibitor, reversed the suppression of cell migration and proliferation caused by MEG3 siRNA and miR-770-5p inhibitor, suggesting that MEG3 and miR-770-5p acted by upregulating SRGAP1. Thus, we speculated that downregulation of MEG3 in accordance with the low-expression of miR-770-5p contributed to the upregulation of SRGAP1, resulting in the suppression of neural cell migration, and leading to the development of HSCR. However, the present study had limitations. Cell functional experiments were not performed in ENCC. Therefore, further research is needed to validate the hypothesis.

In conclusion, the present study showed that MEG3 plays a role in the pathogenesis of HSCR through miR-770-5p and its downstream target SRGAP1. The MEG3/miR-770-5P/SRGAP1 pathway provides a new approach for understanding the etiology of HSCR.

## MATERIALS AND METHODS

### Ethics statement and patient tissue sample

The study was done under compliance with the government policies and the Helsinki Declaration. Moreover, the Institutional Ethics Committee of Nanjing Medical University approved this study. A total of 192 human colon tissues including 96 HSCR patient specimens and 96 matched controls were collected in Nanjing Children’s Hospital Affiliated to Nanjing Medical University from October 2009 to October 2014 after surgery. All HSCR patients, who were diagnosed by barium enema and anorectal manometry evaluation before operative treatment, were made pathological diagnosis to verify that there were no ganglionic cells in their colon tissues after surgery. 96 control samples were obtained from patients who also had acquired surgery and were verified without HSCR or other congenital malformations. Informed consents from their guardians were acquired after full explanations of the study, and the tissues were immediately frozen and stored at −80°C after operation.

### RNA isolation and qRT-PCR

Trizol reagent (Invitrogen Life Technologies Co, CA, USA) was used to extract Total RNA from colon tissues and cells according to manufacturer’s instructions. qRT-PCR was used to detect the expression levels of mRNA, lncRNA and microRNA in tissues and cells. The expression levels of hsa-miR-770-5p in tissues and cells were measured by TaqMan® MicroRNA Assays (Applied Biosystems, CA, USA) with a normalized control. At the same time, qRT-PCR was performed to detect mRNA and lncRNA expression levels on the ABI 7900HT (Applied Biosystems, CA, USA), along with GAPDH as an endogenous control.

### Protein extraction and western blot

To extract proteins, tissues and cells were lysed by RIPA buffer containing protease inhibitors (cOmplete, ULTRA, Mini, EDTA-free, EASYpack Roche, Germany). And then BCA method (Beyotime, Nantong, China) was applied to test protein concentration. In 12% sodium dodecyl sulphate polyacrylamide gel electrophoresis (SDS-PAGE), equal amount of proteins (80μg) were separated, and then transferred to PVDF membrane (Roche Germany). After blocking the proteins in PVDF membrane in 5% skimmed milk. The primary antibody against SRGAP1 was hatched in 4°C overnight, and the second antibody (Beyotime, Nantong, China) which were anti-rabbit HRP-linked was incubated 1 hour in indoor temperature. Finally, ECL reagent (Millpore, MASS, USA) was used to develop the blots. We used ImageJ software to quantify the integrated density of the band, and analyzed the western blot data by Graphpad. Additionally, GAPDH antibody was acted as an internal control.

### Cell culture and cell transfection

Human 293T and SH-SY5Y cell lines which were cultured in complete growth medium:DMEM (Hyclone, UT, USA), supplemented with 10% fetal bovine serum (10%FBS), 100 U/mL penicillin, and 100 μg/mL streptomycin, were obtained from American Type Culture Collection (ATCC, Manassas VA, USA) under the condition of 37°C and 5% CO_2_. In transfection experiments, synthetic miRNA precursor molecules of miR-770-5p, negative control and siRNA of SRGAP1 and MEG3 (GenePharma, Shanghai, China) were used with Lipofectamine 2000 Reagent (Invitrogen, CA, USA) following the protocol of the manufacturer.

### Establishment of stable MEG3 overexpression in cell lines

Cells were plated in 6-well at a concentration of 1.5×10^5^ cells/well and then infected with 1ml MEG3-GFP lentivirus vectors (MEG3 overexpression group) and 1ml GFP lentivirus vectors (negative control group) using 8μl polybrene serum-free medium according the manufacturer’s instructions for 6 hours. After 6 hours, 1ml serum-free medium was adding each well. After 24 hours, Cells were embedded in 10% fetal bovine serum medium for 3 days. GFP expression was examined by fluorescence microscopy (Olympus, Tokyo, Japan). 4 days after infection, cells over-expressed MEG3 stably were used for subsequent experiments. MEG3-GFP lentivirus vectors and GFP lenivirus vectors were donated by Liver Transplantation Center of the First Affiliated Hospital and State Key Laboratory of Reproductive Medicine, Nanjing Medical University.

### Dual-luciferase reporter assay

In the Dual-luciferase reporter assay, the wild-type and mutated 3’-UTR sequence of SRGAP1 mRNA were inserted into the KpnI and SacI sites of pGL3 promoter vector (Genscript, Nanjing, China). We transfected with pRL-SV40, negative control, miR-770-5p mimics, pGL3-SRGAP1 and pGL3-SRGAP1-mut by using Lipofectamine 2000 (Invitrogen Corp, CA, USA). After collection, cells were assessed by using the Dual Luciferase Assay (Promega, Madison, WI) after 48 h transfection following the manufacturer’s instructions. All experiments were repeated three times independently.

### Cell transwell assays and cell proliferation

To estimate the competence of cell migration, cell transwell assays were designed, and executed principally with the application of the Transwell migration chambers (8 μm pore size, Millipore Corporation, Billerica, MA). Firstly, cells were cultured on 6-well plates and transfected with miR-770-5p inhibitor/mimics, SRGAP1 siRNAs, MEG3 siRNAs, MEG3-overexpression lentivirus and negative control. Cells were suspended with serum-free medium, then in the upper chamber 100μl cell suspension was seeded, and in the lower chamber 600ul medium with 10% FBS. After incubated for 24 to 48 hours, cells were dyed with crystal violet staining solution (Beyotime, Nantong, China). We used Image-pro Plus 6.0 to photograph under 40×magnification (five views per well) and counted migrated cells while cell numbers of normal control were normalized to 1. Besides, CCK8 assay (Beyotime, Nantong, China) was applied to test the cell proliferation. The TECAN infinite M200 Multimode microplate reader (Tecan, Mechelen, Belgium) was used to measure the absorbance at 450 nm. All experiments were repeated three times independently.

### Cell cycle and cell apoptosis

For detection of cell cycle assay, cells, which were transfected with miR-770-5p inhibitor, MEG3 siRNA and infected MEG3-overexpression lentivirus, were harvested and detected by BD Biasciences FACS Calibur Flow Cytometry (BD Biasciences, NJ, USA). For cell apoptosis analysis, cells were stained with the Annexin V-FITC/Propidium Iodide Kit (KeyGen Biotech, Nanjing, China) after collection on the basis of the manufacturer’s instructions. Data were analyzed with FlowJo V7 software V7 software (Tree Star, Oregon, USA). All experiments were performed in triplicate independently.

### Statistical analysis

Statistical analysis was performed using STATA9.2 (Stata Corp, Texas, USA), and presented with Graph PAD prism software. Experimental data of tissue samples are analyzed by Wilcoxon rank-sum test to evaluate differences between groups, while the data of cell samples are presented as meank-su from three separate experiments in triplicates per experiment by double-sided Student's t-test. Results were considered statistically significant at *P*<0.05.

## SUPPLEMENTARY MATERIALS FIGURE


