# Correction: Downregulation of lncRNA MEG3 and miR-770-5p inhibit cell migration and proliferation in Hirschsprung’s disease

**DOI:** 10.18632/oncotarget.27049

**Published:** 2019-07-09

**Authors:** Hongxing Li, Bo Li, Dongmei Zhu, Hua Xie, Chunxia Du, Yankai Xia, Weibing Tang

**Affiliations:** ^1^ Department of Pediatric Surgery, Children’s Hospital of Nanjing Medical University, Nanjing, China; ^2^ State Key Laboratory of Reproductive Medicine, Institute of Toxicology, School of Public Health, Nanjing Medical University, Nanjing, China; ^3^ Key Laboratory of Modern Toxicology, Nanjing Medical University, Ministry of Education, Nanjing, China; ^4^ Department of Hepatobiliary Surgery, The Second Affiliated Hospital of Nantong University, Nantong, China

**This article has been corrected:** In Figure 3, the transwell pictures of the Control and miR-770-5p inhibitor treated group were misused in the SH-SY5Y cell lines. The pictures were accidentally arranged together and the wrong transwell picture was copied. After repeated verification of the transwell pictures, we found that in the Figure 4A and Supplementary Figure 1, the picture of the cell cycle was also incorrect. The corrected Figure 3, 4 and Supplementary Figure 1 are shown below. The authors declare that these corrections do not change the results or conclusions of this paper.

Original article: Oncotarget. 2017; 8:69722–69730. 69722-69730 . https://doi.org/10.18632/oncotarget.19207

**Figure 3:**
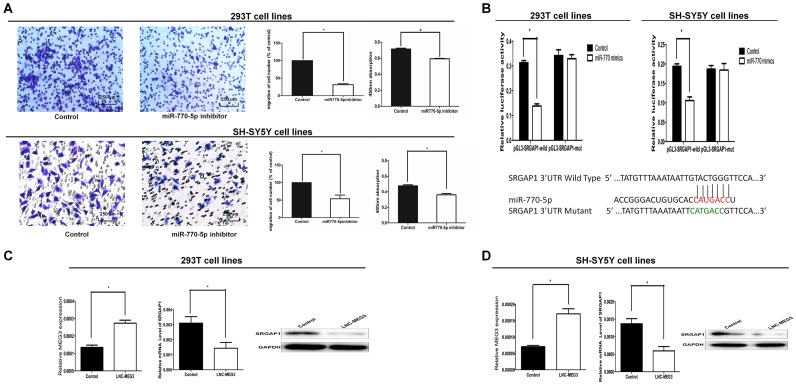
SRGAP1 is a downstream target of MEG3/miR-770-5p. (**A**) Cell migration and proliferation were significantly decreased in 293T and SH-SY5Y cells transfected with the miR-770-5p inhibitor. (**B**) Bottom: Sequence alignment of human miR-770-5p with 3’-UTR of SRGAP1. Mutations in the 3’-UTR of SRGAP1. TOP: The firefly luciferase activity in 293T and SH-SY5Y cells after cotransfection with reporter construct and miR-770-5p mimics. (**C**, **D**) The mRNA and protein expression of SRGAP1 was determined by qRT-PCR and Western blot after transfected with MEG3-overexpression lentivirus in 293T and SH-SY5Y cells. All results were presented as mean ± SE.* means significant difference (P < 0.05).

**Figure 4:**
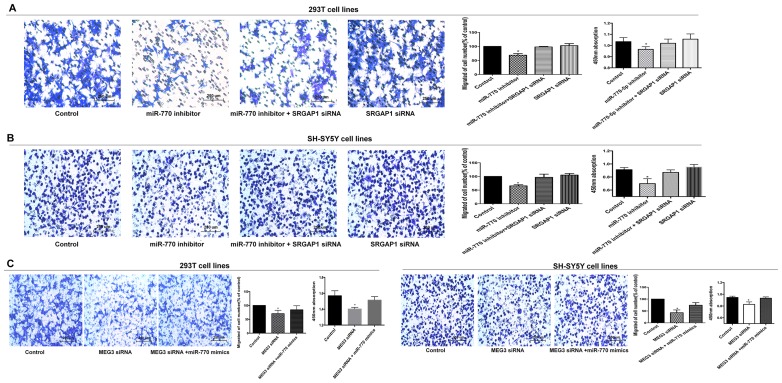
MEG3 effect on cell migration and proliferation was mediated by the miR-770-5p/SRGAP1 pathway in HSCR. (**A**, **B**) SRGAP1 siRNA could partly reverse cell migration and proliferation in 293T and SH-SY5Y cell lines transfected with miR-770-5p inhibitor. (**C**) Co-transfection of miR-770-5p mimics partially rescued the MEG3 siRNA-mediated decrease in cell migration and proliferation. Absorbance at 450 nm was presented as mean ± SE. ^*^means significant difference (P < 0.05).

**Supplementary Figure 1:**
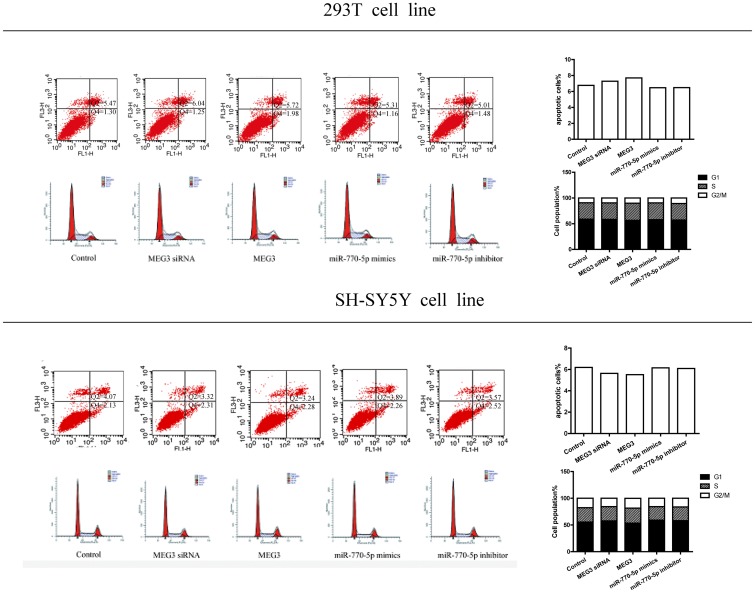
The results of cell cycle and apoptosis. The results of apoptosis and cell cycle of MEG3 siRNA, MEG3, miR-770-5p mimics and miR-770-5p inhibitor treated groups in 293T cell lines and SH-SY5Y cell line. The data showed that there was no obvious difference in the number of apoptotic cells (%) and cell population between these groups.

