# Supplementary material for: Downregulation of lncRNA MEG3 and miR-770-5p inhibit cell migration and proliferation in Hirschsprung’s disease
Source: Oncotarget. 2017 Jul 12;8(41):69722–30. doi: 10.18632/oncotarget.19207 (PMC5642511; doi:10.18632/oncotarget.19207)
Supplement: Supplementary file 1 [file oncotarget-08-69722-s001.pdf]

Downregulation of lncRNA MEG3 and miR-770-5p inhibit cell migration and proliferation in Hirschsprung's disease

SUPPLEMENTARY MATERIALS

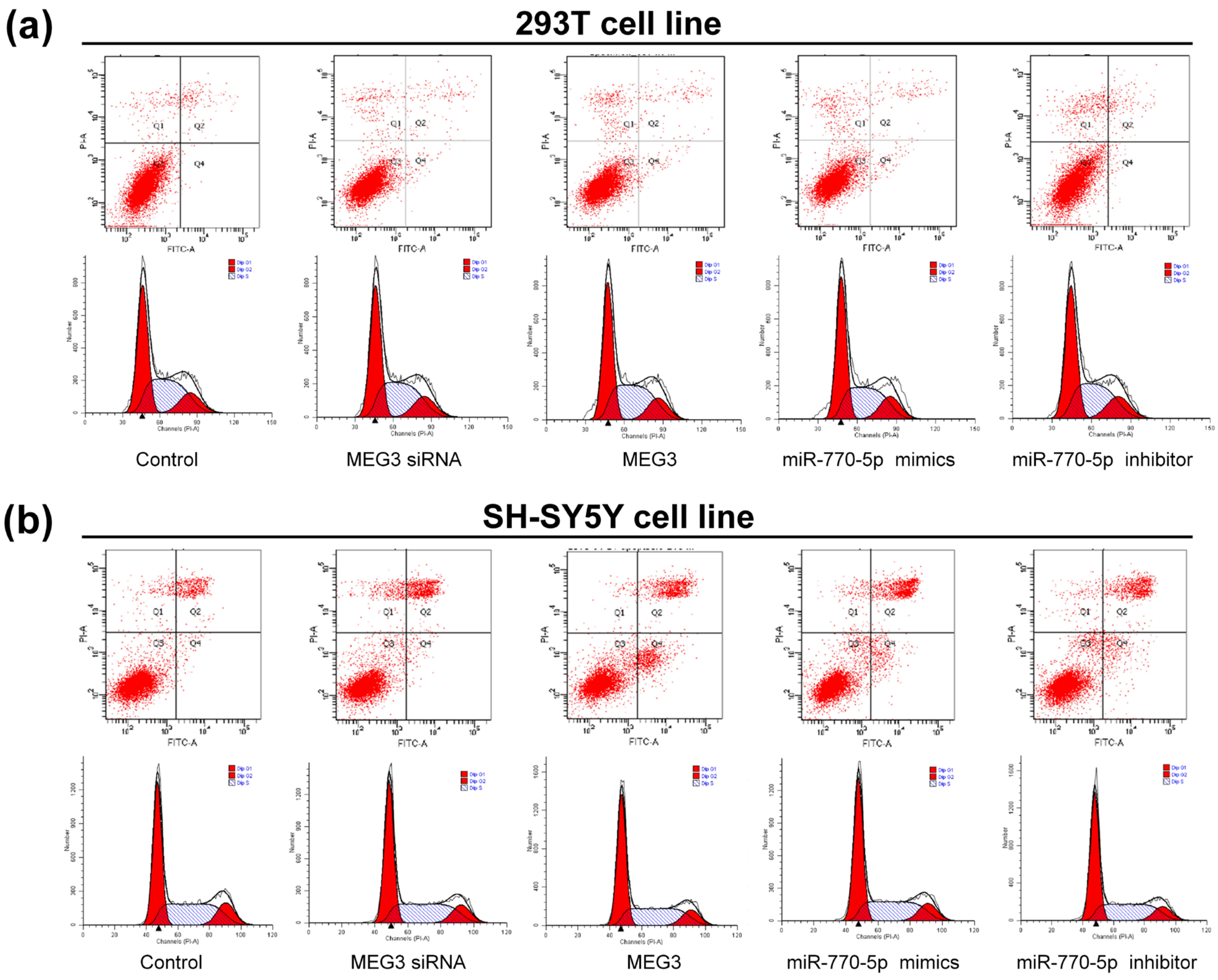

Supplementary Figure 1: The results of cell cycle and apoptosis. (a) The results of apoptosis and cell cycle of MEG3 siRNA, MEG3, miR-770-5p mimics and miR-770-5p inhibitor treated groups in 293T cell lines. **(b)** The same works in SH-SY5Y cell line.
